# Supplementary material for: How Does Pruning Impact Long-Tailed Multi-Label Medical Image Classifiers?
Source: ArXiv. 2023 Aug 17:arXiv:2308.09180v1. Preprint. [Version 1] (PMC10543014)
Supplement: 1 [file NIHPP2308.09180V1-supplement-1.pdf]

Supplementary Materials

Table 1: Summary of implementation details for training. LR = learning rate.

**Model**	ResNet50 (ImageNet-pretrained)
**Optimizer (LR)**	Adam (1×10^{-4})
**Augmentations**	Random horizontal flip, Random rotation (-15°, 15°)
**Preprocessing**	ImageNet normalization, Resize to 256 × 256
**Early Stopping**	15 epochs with no improvement in validation AUC
**Framework**	PyTorch
**Hardware**	1 NVIDIA RTX A6000 GPU
**Training Walltime**	~3 hours (NIH-CXR-LT), ~6.5 hours (MIMIC-CXR-LT)

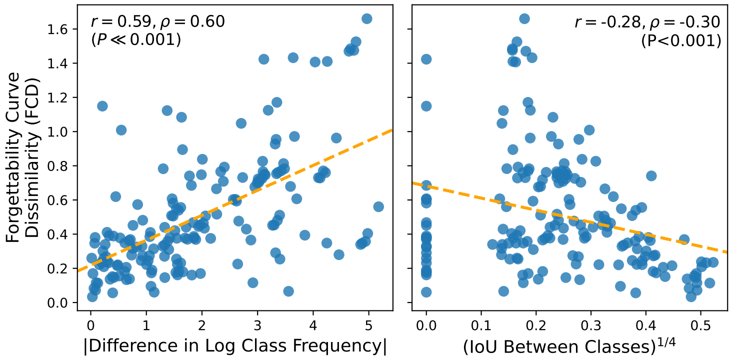

Fig. 7: Mutual relationship between pairs of diseases and their forgettability curves. For each pair of MIMIC-CXR-LT classes, FCD is plotted against the absolute difference in log frequency (left) and IoU between the two classes (right). Pearson (r) and Spearman (ρ) correlation coefficients shown, with P-value for ρ.

Table 2: Long-tailed distribution of NIH-CXR-LT and MIMIC-CXR-LT labels by data split. Italicized labels with an asterisk before the name denote the five newly added rare disease findings.

Label	NIH-CXR-LT				MIMIC-CXR-LT			
	Train	Val	Test	**Total**	Train	Val	Test	**Total**
No Finding	44625	6766	8015	59406	34380	3768	9828	47976
Support Devices	-	-	-	-	73641	8328	22397	104366
Lung Opacity	-	-	-	-	57591	6397	17405	81393
Infiltration	12739	1996	5159	19894	-	-	-	-
Atelectasis	7587	1272	2700	11559	51055	5595	15102	71752
Effusion	7919	1663	3735	13317	53029	5822	16300	75151
Nodule	4359	667	1305	6331	-	-	-	-
Mass	3689	764	1329	5782	-	-	-	-
Pneumothorax	2432	764	2106	5302	11579	1388	3517	16484
Consolidation	2626	544	1497	4667	12406	1413	3776	17595
Cardiomegaly	1590	318	868	2776	55020	6026	16747	77793
Pleural Thickening	1998	485	902	3385	-	-	-	-
Fibrosis	1138	183	365	1686	-	-	-	-
Edema	1283	269	751	2303	30555	3502	9530	43587
Emphysema	1327	272	917	2516	-	-	-	-
Pneumonia	806	173	452	1431	32131	3589	9455	45175
**Subcutaneous Emphysema*	957	221	813	1991	1900	269	599	2768
Enlarged Cardio -mediastinum	-	-	-	-	19880	2188	6173	28241
Fracture	-	-	-	-	7823	862	2410	11095
Lung Lesion	-	-	-	-	1707	172	526	2405
**Tortuous Aorta*	598	49	95	742	2212	233	641	3086
**Calcification of the Aorta*	368	32	55	455	2595	282	811	3688
**Pneumo-peritoneum*	214	33	69	316	448	59	134	641
**Pneumo-mediastinum*	88	22	143	253	571	87	195	853
Hernia	130	35	62	227	-	-	-	-
Pleural Other	-	-	-	-	373	42	138	553
**Total**	78506	12533	21081	112120	182386	20363	54269	257018
